# Development of Glycerosomal pH Triggered In Situ Gelling System to Ameliorate the Nasal Delivery of Sulpiride for Pediatric Psychosis

**DOI:** 10.3390/gels10090608

**Published:** 2024-09-23

**Authors:** Mona M. Shahien, Alia Alshammari, Somaia Ibrahim, Enas Haridy Ahmed, Hanan Abdelmawgoud Atia, Hemat A. Elariny, Marwa H. Abdallah

**Affiliations:** 1 Department of Pediatrics, College of Medicine, University of Ha’il, Ha’il 81442, Saudi Arabia; m.shahin@uoh.edu.sa (M.M.S.); so.bashir@uoh.edu.sa (S.I.); 2 Department of Pharmaceutics, College of Pharmacy, University of Ha’il, Ha’il 81442, Saudi Arabia; alia.alshammari@uoh.edu.sa; 3 Department of Anatomy, College of Medicine, University of Ha’il, Ha’il 81442, Saudi Arabia; e.haridy@uoh.edu.sa; 4 Department of Anatomy and Embryology, Faculty of Medicine, Ain Shams University, Cairo 11566, Egypt; 5 Department of Pharmacology and Toxicology, College of Pharmacy, University of Ha’il, Ha’il 81442, Saudi Arabia; h.soliman@uoh.edu.sa (H.A.A.); hem.mohammed@uoh.edu.sa (H.A.E.); 6 Department of Biochemistry, Faculty of Pharmacy, Al-Azhar University, Cairo 11651, Egypt; 7 Department of Pharmacology and Toxicology, Faculty of Pharmacy, Al-Azhar University, Cairo 11651, Egypt; 8 Department of Pharmaceutics, Faculty of Pharmacy, Zagazig University, Zagazig 44519, Egypt

**Keywords:** sulpiride, glycerosomes, in situ intranasal gel, ex vivo permeation, bioavailability studies

## Abstract

Sulpiride (Sul) is a medication that blocks dopamine D_2_ receptors. It is used to treat gastrointestinal disturbances and has antipsychotic effects depending on the dose given. Sulpiride is subject to P-glycoprotein efflux, resulting in limited bioavailability and erratic absorption. Hence, the aim of this study was to generate a glycerosomal in situ gel of sulpiride for intranasal administration, specifically targeting children with schizophrenia who may have difficulty swallowing traditional solid medications, for enhancing its bioavailability. This study aimed to demonstrate the efficacy of intranasal administration of glycerin-encapsulated lipid-nanovesicles (glycerosomes) mixed with in situ gels for prolonged release of anti-psychotic medication. A Box–Behnken design was utilized to create sulpiride-loaded glycerosomes (Sul-GMs), with the lipid amount (A), glycerin concentration (B), and sonication time (C) acting as independent variables. Their impact on the entrapment efficiency, EE% (Y_1_), and in vitro drug release (Y_2_) were evaluated. The sulpiride EE% showed an increase when the glycerin concentration was raised to 25% *v*/*v*. Nevertheless, when the glycerin concentration was raised to 40% *v*/*v*, there was a notable decrease in the EE%. The optimized glycerosome was added to pH triggered carbopol 974P in situ gel formulations including HPMC K15M with different concentrations. The in situ gel formulation (G3) comprising 0.6% carbopol 974P and 0.6% hydroxypropyl methyl cellulose-K15M (HPMC K15M) demonstrated suitable pH, viscosity, desired gel strength, spreadability, and mucoadhesive strength. Consequently, it was selected for in vitro study, ex vivo permeation investigation, and in vivo evaluations. The glycerosomal in situ gel exhibited favorable ex vivo permeability of SU when applied to the nasal mucosa. The pharmacokinetic investigation revealed that the optimized Sul-loaded glycerosomal in situ gel exhibited a significant fourfold and twofold enhancement in systemic bioavailability compared to both the control gel and the commercially available formulation. Finally, the intranasal administration of Sul-loaded glycerosomal in situ gel is a promising alternative to oral treatment for pediatric patients with psychosis.

## 1. Introduction

Sulpiride (Sul) is classified as a pharmacological compound that exhibits selective inhibition of dopaminergic receptors and has been identified as highly efficacious for pediatric formulation [1,2]. Sulpiride is utilized as a neuroleptic agent in the treatment of a range of central nervous system (CNS) disorders, including schizophrenia, depression, and psychosis. Additionally, it is employed in the management of gastrointestinal tract (GIT) disturbances such as emesis and dyspepsia, with the specific effects dependent on the dosage supplied [1]. It exhibits physicochemical properties that indicate a poor solubility in water and restricted permeability. As a result, it is categorized as a Class IV drug according to the biopharmaceutical classification system [3]. Therefore, a previously reported oral bioavailability of 27% has been defined [4]. It is important to develop a strategy that enhances its bioavailability by addressing challenges such as limited solubility/dissolution rate, low permeability, and P-gp efflux. Therefore, many approaches have been explored to enhance the bioavailability of sulpiride, such as solid dispersion, microemulsion, nanoparticles [2,5,6], and nanoliposphers [7].

Glycerosomes (GMs) are a newly developed type of flexible nanovesicles. They offer a novel approach to modifying vesicle fluidity, providing distinct advantages over traditional vesicular carriers such as liposomes. Glycerosomes are composed of a mixture of different phospholipids, together with 20–30% (*v*/*v*) of glycerin that is not harmful with or without cholesterol. The presence of glycerin enables them to possess enough deformability and flexibility to enter pores that are considerably smaller than their own dimensions [8]. They have been employed in many research studies to facilitate the transdermal, pulmonary, and intranasal administration of a wide range of medications [9,10,11].

Pediatric dosage forms that are commercially accessible, like pills, capsules, and oral liquids, have several drawbacks. Like geriatrics, pediatric patients may face difficulties swallowing traditional oral dosage forms, which could lead to noncompliance with the recommended treatment plan [12,13]. As a result, new developments in formulation technology aim to improve drug safety and efficacy by creating an appropriate dosage form that will improve patient compliance. Consequently, to improve patient compliance and the pharmacological effect of sulpiride in both geriatrics and pediatric patients, sulpiride-loaded glycerosomes were incorporated into in situ gelling agents for nasal delivery.

Drug delivery through nasal administration is one of the biggest challenges facing pharmaceutical scientists. Nasal delivery is markedly efficacious when oral administration of a medicine yields an undesired adverse reaction [14]. Intranasal drug delivery systems have several advantages, such as the capacity to bypass hepatic first-pass metabolism, hence improving drug bioavailability and offering a noninvasive, efficient route for drug administration. Moreover, intranasal administration has shown promise as an effective approach to bypass the blood–brain barrier (BBB), allowing for direct delivery of medications that operate on the central nervous system (CNS) [15]. 

Consequently, multiple approaches have been developed to improve the intranasal administration of medicinal agents through the use of nano-based carriers, including nanostructured lipid carriers [16], nanoparticles [17], liposomes [18], ethosomes [19], and nanoemulsion [20].

An important point during the manufacturing of nasal drug delivery is to overcome the protective barriers of the nasal cavity without inducing lasting tissue harm. Hence, an alternative approach is to reduce the mucociliary clearance by employing mucoadhesive gel formulations. This will extend the duration of the drug’s presence at the nasal absorption site, thereby enhancing its absorption. In order to increase nasal bioavailability, approaches that use in situ gelling polymers or those that increase viscosity try to extend the duration of time that the drug is in contact with the nasal surface. In situ gelling systems possess favorable characteristics that make them suitable for drug delivery. They are minimally invasive and can be used to achieve greater concentrations of drug at the intended site of action, while minimizing systemic side effects. Additionally, they are biodegradable, biocompatible, and enable sustained release over a prolonged duration, thus improving patient adherence [21]. These features make them suitable for delivering drugs intranasally. The in situ gel systems exist as a free flowing liquid at room temperature but undergo a sol-to-gel phase transition in response to precise changes in physicochemical parameters, such as ionic concentration, temperature, or pH, when they come into contact with body fluids. Accordingly, there are three commonly accepted forms of in situ gelling systems that can generate a sol-to-gel phase: temperature sensitive systems, pH triggered systems, and ion activated systems [22].

As far as we are aware, no research has been conducted using glycerosomal in situ gel to increase sulpiride’s bioavailability. The novelty of our investigation resides in exploring the ability to generate an intranasal nanovesicular preparation of sulpiride as an innovative drug delivery system, with a special focus on its ex vivo permeability and bioavailability. Hence, the objective of this study was to augment the transmucosal permeation of sulpiride to achieve increased therapeutic efficacy of the drug through intranasal delivery. To investigate the impact of various variables on the characteristics of glycerosomal systems using the Box–Behnken design (BBD), a number of Sul-loaded glycerosomes-based systems were developed. The best Sul-loaded glycerosomes were then added to the in situ gelling system, then physicochemical and ex vivo permeation investigations were conducted. Lastly, the possibility of enhancing the transmucosal permeability of Sul and improving its antipsychotic effectiveness was examined using in vivo pharmacokinetic investigations of the optimized Sul-loaded glycerosomal in situ gel formulation following intranasal application into rats.

## 2. Results and Discussion

### 2.1. Statistical Design of Sulpiride-Loaded Glycerosomes

#### 2.1.1. Box–Behnken Design (BBD) Analysis

This study aimed to evaluate the correlation between various independent variables, namely, the quantity of phospholipid (A), glycerin concentration (A), and time of sonication (C), with the corresponding dependent responses, percentage of encapsulation efficiency (Y_1_) and the percentage of in vitro drug release after 6 h (Y_2_). The Box–Behnken design was employed for this purpose, utilizing the Design Expert^®^ software. For two dependent responses, it was concluded that the quadratic model was the best one. The significance of the quadratic model in describing the correlation between the independent factors and dependent answers was confirmed by the *p*-value being less than 0.05. Depending on the findings of 3^3^ BBD experiments, it was observed that the quantities of phospholipid (lipid producing vesicles), the concentration of glycerin, and the duration of sonication had a notable influence on both the entrapment efficiency and the percent of in vitro drug release. The statistical significance of the model was assessed using ANOVA, with a *p*-value of less than 0.05 indicating that the model is regarded to be statistically significant. 

#### 2.1.2. Influence of the Formulation Factors on the Encapsulation Efficiency (Y_1_)

The entrapment efficiency (EE%) of the generated glycerosomal formulations ranged from 38.87 ± 0.71% to 79.32 ± 1.00%, as presented in Table 1. These results demonstrate the successful encapsulation of the medication in glycerin-containing nanovesicles, indicating the potential of utilizing these nanovesicles as an effective delivery vehicle for sulpiride. The data on EE% were most effectively analyzed using a quadratic model, with *p*-values less than <0.0001 indicating statistical significance. The adequate precision of the analysis was found to be high, with a value of 60.0489. Furthermore, a difference of less than 0.2 was observed between the adjusted R^2^ (0.9948) and the predicted R^2^ (0.9743). The equation provided below demonstrates a correlation between the factors under investigation and the percentage of EE.

Y_1_ = 65.50 + 9.89 A+ 3.68 B − 1.90 C + 5.76 AB − 5.47 AC − 2.24 BC − 1.91 A^2^ − 13.41 B^2^ − 1.87 C^2^

**Figure 1:**
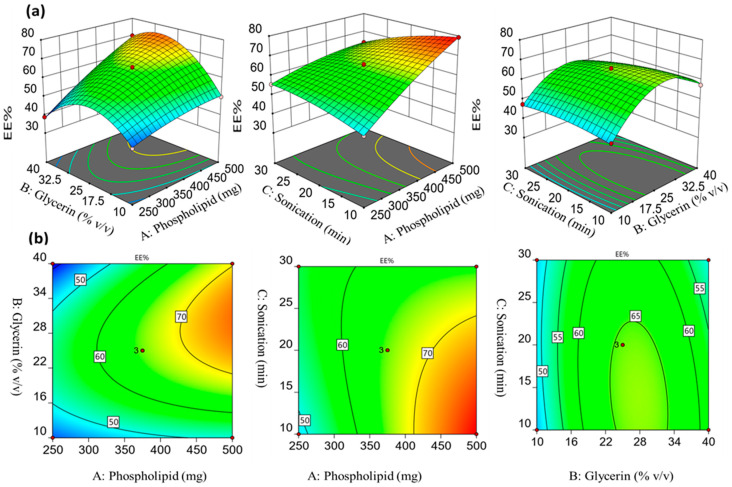
Response surface (**a**) and corresponding contour graphs (**b**) representing the influences of the formulation factors on the EE% (Y_1_).

The 3D response surface graphs and corresponding contour plots depict the effects of independent parameters on the EE% of sulpiride-loaded glycerosomes. As depicted in Figure 1, the amount of phospholipid had a notable impact on the EE% of sulpiride in the glycerosomal vesicles. The positive coefficient associated with factor A indicates that there was a positive relationship between the EE% and the amount of phospholipid. The formulation number F2, which consisted of 500 mg of phospholipid, exhibited an encapsulation efficiency (EE%) of 79.32 ± 1.00%. In comparison, F4, containing 250 mg of phospholipid, had an EE% of 47.98 ± 0.50%. The observed correlation between the EE% and the phospholipid amount may be attributed to the lipophilicity of sulpiride, which tends to have a higher affinity for the lipid-rich surface area, leading to its entrapment within the vesicles lipid phase [23,24,25].

As depicted in Figure 1, increasing the glycerin concentration (% *v*/*v*) from 10 to 25% exhibited a comparable elevation in the EE% of sulpiride. However, increasing the glycerin concentration to 40% *v*/*v* led to a significant drop in the EE%. A low concentration of glycerin led to an increase in the particle size which led to the increase in EE%, while a further increase in the glycerin concentration could improve the drug solubility, causing a possible leakage of drug and decrease in EE% [23]. These findings are aligned with the previous research conducted by Younis and Habib, which showed that the higher concentration of glycerin had an adverse effect on the entrapment process of sertaconazole nitrate-loaded glycerosomes [8]. Furthermore, prolonging the duration of sonication resulted in a proportional reduction in the entrapment efficiency %. This phenomenon can be related to the reduction in the glycerosomal particle size that occurs as the sonication time is increased [26].

#### 2.1.3. Influence of the Formulation Factors on the Drug Release % after 6 h (Y_2_)

**Figure 2:**
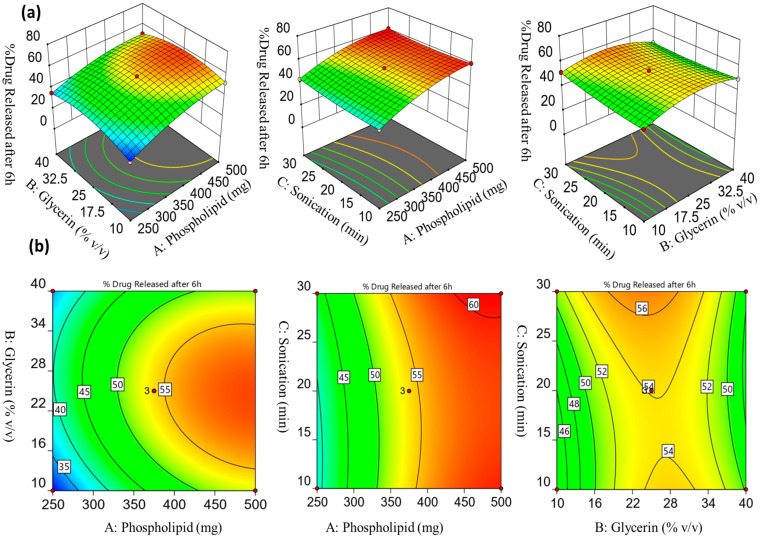
Response surface (**a**) and corresponding contour graphs (**b**) representing the influences of the formulation factors on percentage drug released after 6 h (Y_2_).

As a result of a *p*-value that was lower than 0.0001, it was decided that the quadratic model was the most suitable for fitting the data on the percentage of drug release. The adjusted R^2^ (0.9952) and predicted R^2^ (0.9755) values exhibited a small difference of less than 0.2, indicating the model’s validity. Additionally, the model demonstrated a high level of adequate precision, with a value of 62.0078. The release of sulpiride from glycerosomes was investigated by an in vitro study conducted at various time intervals for 6 h. As shown in Table 1, it was reported that F3, which had a high amount of phospholipid (500 mg), demonstrated the highest drug release after six hours (60.94 ± 0.86%); on the other hand, F6, which had a low amount of lipid-producing vesicles, demonstrated low drug release (43.36 ± 2.21%). The concentration of glycerin, ranging from 10 to 25% *v*/*v*, was found to have a direct impact on the drug release from glycerosomes after a six-hour period, while maintaining a constant concentration of phospholipid. The drug release percentage for formulation F15, consisting of 500 mg of phospholipid and 10 mg of glycerin, was found to be 51.80 ± 0.75. In comparison, the drug release % for formulation F3, comprising 500 mg of phospholipid and 25 mg of glycerin, was 60.94 ± 0.86. An additional increase in the concentration of glycerin to 40% led to a discernible reduction in the proportion of medication released (50.91 ± 1.97; F10). The reduced drug release observed at lower glycerin concentration may be attributable to the increased structural integrity and reduced permeability of the lipid membranes of glycerosomal vesicles, hence impeding drug release. Furthermore, at high glycerin concentration (40% *v*/*v*), the amount of sulpiride released was found to be limited. This can be attributed to the lower efficiency of drug entrapment at high glycerin concentration. The 3D response surface and the related contour graphs (Figure 2) provide evidence for our findings regarding the influence of formulation variables A, B, and C on Y_2_. Moreover, the correlation between the dependent variables and their respective independent variables can be confirmed through the utilization of the polynomial equation, which was obtained from the Box–Behnken design and is presented in the following manner:

Y_2_ = 54.07 + 9.55 A + 0.77 B + 1.35 C − 1.63 AB − 0.91 AC − 2.05 BC − 4.84 A^2^ − 7.27 B^2^ + 1.53 C^2^

#### 2.1.4. Validation and Optimization Technique

**Figure 3:**
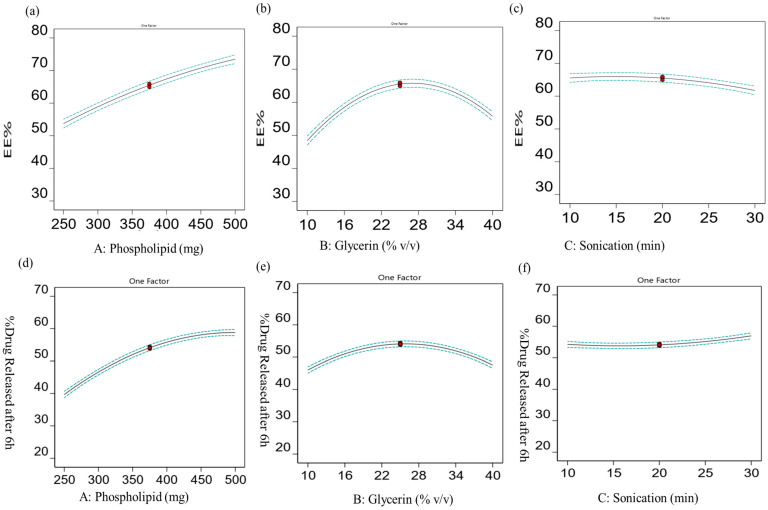
Box–Behnken design plots showing the influence of (**a**) lipid amount on EE%, (**b**) glycerin concentration on EE%, (**c**) sonication time on EE%, (**d**) lipid amount on % drug release, (**e**) glycerin concentration on % drug release, and (**f**) sonication time on % drug release.

It was observed that the lack of fit in the responses of the dependent variables Y_1_ and Y_2_ resulted in insignificant F-values of 3.47 and 4.28, and *p*-values of 0.1951 and 0.2320 for Y_1_ and Y_2_, respectively (*p* > 0.05). This indicates that the model can be considered valid. Figure 3a–f show the statistical plot illustrating the correlation between the actual and anticipated values for the responses, as well as their residual plot. The optimum formulation with the desired properties was determined through the implementation of the BBD and the point-prediction method. The optimized formula was chosen by a selection process involving 15 experiments. The parameters for selection were adjusted to prioritize the attainment of maximum values for the two variables, Y_1_ and Y_2_. The study revealed that, the glycerosomal formulation, consisting of 497 mg of phospholipid and 31.13% *v*/*v* of glycerin, demonstrated compatibility with the desired criteria for an optimal formulation when prepared at 11.61 min of sonication. It showed predicted values of 80.72 ± 0.84% and 58.61± 0.61% for Y_1_ and Y_2_, respectively. The predicted values were in accordance with the observed values (77.86 ± 1.24% and 61.83 ± 1.56% for Y_1_ and Y_2_, respectively). The findings of this investigation show that the optimization procedure was effective in generating glycerosomes loaded with sulpiride using the Box–Behnken design.

### 2.2. Evaluation of the Optimized Sulpiride-Loaded Glycerosomal Formulation

#### 2.2.1. Vesicle Size, Zeta Potential Determination, and TEM Analysis

**Figure 4:**
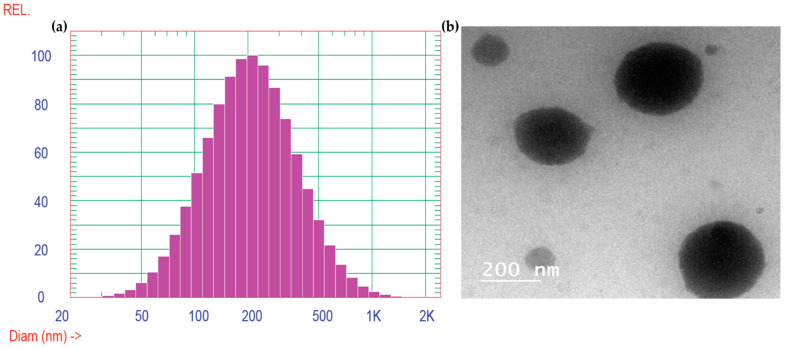
Vesicles size distribution curve of optimized sulpiride-loaded glycerosomal formulation (**a**) and transmission electron micrograph (**b**).

Transmission electron microscopy (TEM) image exhibits the presence of tiny, smooth, and round vesicles, exhibiting particle sizes that were found to be similar to those produced by the application of the dynamic light scattering approach, as illustrated in Figure 4. The dynamic light scattering (DLS) study was conducted to determine the vesicle size and the polydispersity index (PDI) of the glycerosomes. The mean vesicle size was determined to be 244.5 nm, with a polydispersity index of 0.353. The PDI value indicates the homogeneity of size distribution. Together, these characteristics provide a comprehensive evaluation of the formulation’s particle size and distribution.

The dispersion did not exhibit any aggregates that demonstrated desirable physical stability [27]. Zeta potential is a quantifiable attribute that may be used to predict the physical stability of the nanovesicular formulations [28]. The zeta potential of the generated optimum preparation (Sul-GMs) was determined to be −21.9 ± 1.8 mV in our investigation. The strong negativity observed indicates that the glycerosomal vesicles were successfully stabilized by the generation of repulsive forces among particles of same charge, hence impeding their aggregation and enhancing their physical stability [29].

#### 2.2.2. Differential Scanning Calorimetry

**Figure 5:**
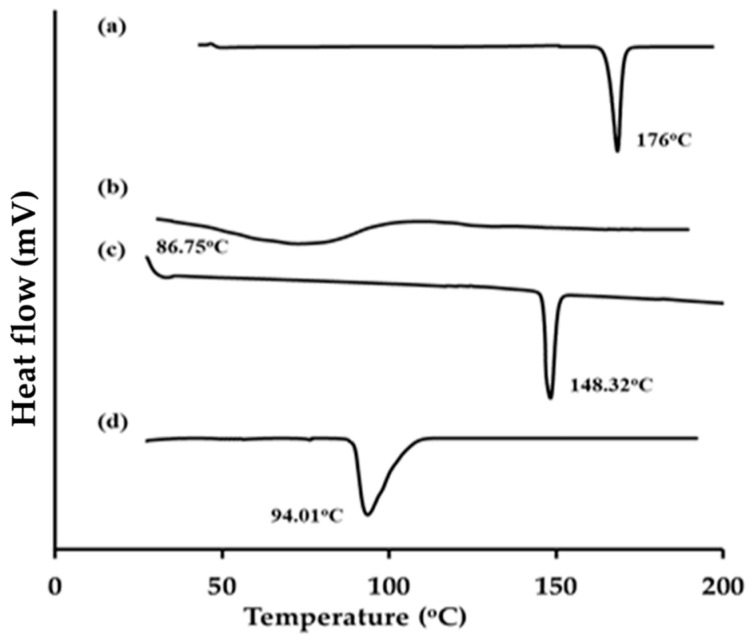
DSC thermograms of (**a**) pure sulpiride, (**b**) soy lecithin, (**c**) cholesterol, and (**d**) optimized sulpiride-loaded glycerosomes.

Figure 5 displays the DSC thermograms of sulpiride, lecithin, cholesterol, and the glycerosomal formula that exhibits optimal characteristics. The experimental results demonstrated that pure sulpiride exhibited a distinct endothermic peak at a temperature of 175 °C, which can be attributed to its crystalline melting point (Figure 5a). The thermal characteristics of soy lecithin and cholesterol exhibited distinct endothermic peaks at 86.75 °C and 148.32 °C, respectively, as illustrated in Figure 5b,c. Furthermore, the optimum glycerosomal formula loaded with sulpiride, as depicted in Figure 5d, demonstrated a wide endothermic peak accompanied by a reduction in intensity at 94.01 °C. Therefore, based on the findings of the study, it can be inferred that there is a significant level of interaction between the drug and bilayer components. This observation suggests successful encapsulation of the drug into the generated glycerosomes. 

### 2.3. In Vitro Release Investigation

**Figure 6:**
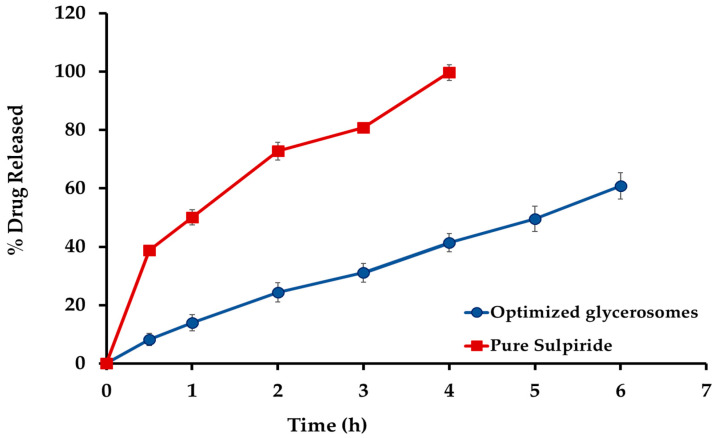
In vitro release of sulpiride from the optimized glycerosomes.

The in vitro release of sulpiride from the optimum glycerosomal formulation is illustrated in Figure 6. Sulpiride solution demonstrated a statistically significant increase in the rate of drug release (*p* < 0.05), with about 99% of the drug being released within four hours. In comparison, during a period of 6 h, a significant proportion of 61.83 ± 3.15% of sulpiride was observed to be released from the optimized sulpiride-loaded glycerosomes. The data unambiguously showed that the glycerosomes exhibited a significant capacity to deliver the medication in a controlled way for a long period. In comparison, the drug solution demonstrated almost complete and quick release of the medication within 4 h. The slower release rate may be due to a proportion of the drug encapsulated into the glycerosomal vesicles, which then would release slowly to the medium through the bilayer membrane. This finding provides confirmation of the glycerosomal capacity to encapsulate sulpiride and limit its release.

### 2.4. Evaluation of pH-Induced Carbopol In Situ Glycerosomal Gel

#### 2.4.1. Physicochemical Evaluation of Carbopol Formulations

Based on the results provided in Table 2, the pH values of all carbopol in situ gel formulations were within the range of 5.8–6.5, which is generally accepted as the appropriate pH range for nasal applications. The pH level mentioned would promote the safe attachment of the gel compositions to the nasal mucosa without causing irritation [30].

#### 2.4.2. In Vitro Gelation and Viscosity of pH-Induced Carbopol Glycerosomal Gel In Situ Gels

Gelation capacity and viscosity are the essential requirements for the development of an in situ gelling system. The formulation should possess an optimal viscosity to facilitate easy administration into the nasal mucosa in the form of liquid droplets. Additionally, it should exhibit a fast transition from a sol state to a gel state, induced by an increase in pH, 6.4. Furthermore, in order to enhance the continuous delivery of sulpiride to the nasal mucosa, it is important that the gel maintains its structural integrity without undergoing erosion or dissolving over an extended duration [31]. The utilization of carbopol for the preparation of in situ gelling systems is supported by the characteristic of its aqueous solutions to undergo a transition into rigid gels upon an increase in pH [32]. Nevertheless, the concentration of carbopol required for the formation of rigid gels leads to the production of strongly acidic solutions, which are not readily neutralized by the buffering capacity of the nasal fluid [33]. The addition of viscosity-enhancing polymers, such as HPMC K15M, may enable a decrease in carbopol concentration while maintaining the gelling capacity and rheological features of the delivery system [34]. To determine the appropriate compositions for utilization of in situ gelling systems, several concentrations of carbopol 974P (0.2–0.6% *w*/*w*) and HPMC (0.2–1.0% *w*/*w*) were assessed for their ability to form gels. According to the data shown in Table 2, it can be observed that the gelling capacity grade labeled as “++” exhibited a higher level of satisfaction.

The viscosity of carbopol formulations exhibited a positive correlation with the concentration of hydroxypropyl methylcellulose (HPMC), as shown in Table 2. The importance of viscosity is essential in the optimization of intranasal formulations as it controls their flow properties, ability to spread, drug release, and the residence duration in the nasal mucosa [31]. On the other hand, gel strength was consistent with the corresponding changes in viscosity (Table 2). The developed formulations should be conveniently delivered in the form of drops and must possess adequate potency to effectively prevent post-nasal drip or anterior leakage. It was found that formulations with gel strength below 25 s exhibited a weak gel structure that had a short duration on application. Consequently, there is a possibility of fast erosion and leakage of drugs in the nasal cavity. Moreover, formulations with a gel strength exceeding 50 s result in the formation of an extremely rigid gel, which can lead to pain. Therefore, a gel strength ranging from 25 to 50 s was deemed ideal for easy application and long-lasting of the dose without any leakage.

#### 2.4.3. Investigation of the Mucoadhesive Strength 

The mucoadhesive strength values of the generated glycerosomal in situ gel formulations were determined and are reported in Table 2. It was concluded that mucoadhesive strength levels between 4000 and 6000 dynes/cm^2^ are suitable for intranasal delivery [35]. Chaudhary and Verma suggest that formulations with sufficient mucoadhesive strength may have the potential to enhance the duration of residence within the nasal cavity [36]. This mechanism can efficiently restrict the elimination of drugs, hence prolonging their release. In contrast, an elevated degree of mucoadhesive strength possesses the capacity to induce harm to the nasal mucosa. The mucoadhesiveness was observed to be positively correlated with the amounts of both carbopol and HPMC. This is due to the fact that high concentration causes the gel structure to become thicker, leading to enhanced interaction between the gel and the mucous membrane through hydrogen bonding [37]. 

#### 2.4.4. Assessment of Spreadability 

The evaluation of spreadability revealed a relationship between spreadability and the viscosity of the in situ gel. By increasing the polymer concentration, the spreadability is reduced. Spreadability is a fundamental attribute that relates to the ability of a gel to distribute and cover a designated surface area upon intranasal application. This property facilitates consistent gel application and ensures that the appropriate dosage of the drug is delivered. This study observed that the spreadability of gel was shown to have an inverse relationship with the viscosity of different in situ gel preparations, as indicated in Table 2. 

Finally, formulation (G3) composed of 0.6% *w*/*w* carbopol and 0.6% *w*/*w* HPMC demonstrated an acceptable pH, viscosity, desirable gel strength, spreadability, and mucoadhesive strength. Therefore, the optimum formulation (G3) including SU-loaded glycerosomes was chosen and subsequently examined for in vivo study, ex vivo permeability investigation, and in vivo evaluations.

### 2.5. In Vitro Drug Release Studies Sul-GMs In Situ Gel Formulation

**Figure 7:**
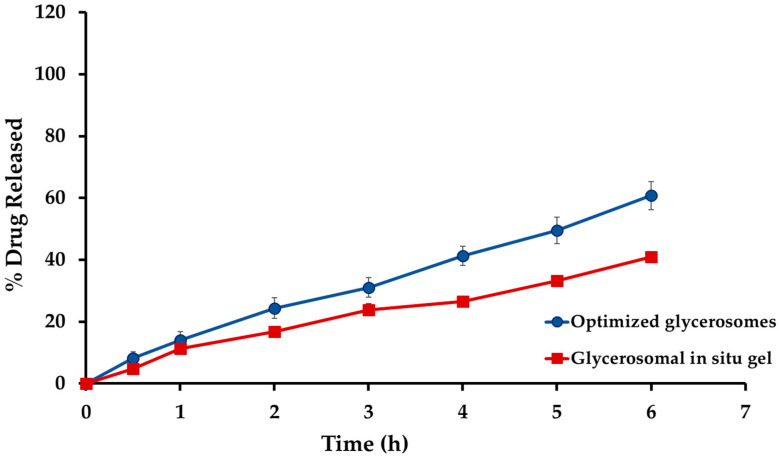
In vitro drug release from sulpiride-loaded glycerosomal in situ gel formulation.

Figure 7 shows the in vitro release patterns of the optimized glycerosome dispersion and the optimized glycerosomal in situ gel formulation. The glycerosomal-based in situ gel exhibited a reduced release rate of sulpiride in vitro, which can be attributed to the higher viscosity caused by the creation of a three-dimensional gel network [38]. Moreover, the decreased rate of sulpiride release noticed in the in situ gel formulations may be attributed to the existence of glycerosomal vesicles that can encapsulate the medication. The vesicles were enveloped with a polymeric covering, resulting in an increased distance for the medication to travel before being released into the surrounding environment. Consequently, the overall release of sulpiride is reduced.

### 2.6. Ex Vivo Permeability Study of the Optimized Sulipride-Loaded Glycerosomal In Situ Gel 

**Figure 8:**
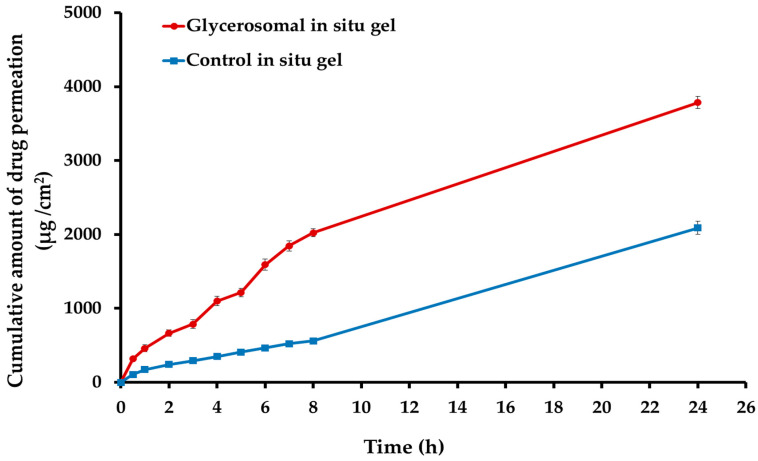
Ex vivo permeation of sulpiride from the optimized glycerosomal in situ gel formulation (G3) and control in situ gel loaded with sulpiride.

The ex vivo permeability study was conducted to evaluate the optimized sulpiride-loaded glycerosomal in situ gel. The findings depicted in Figure 8 illustrate that the inclusion of Sul into the glycerosomal in situ gel (G3) resulted in a notable improvement in the permeation of Sul across the nasal mucosa in comparison to the control in situ gel (consisting of the same gel composition of G3 containing pure Sul powder). Following a 6 h period, the optimized in situ gel containing Sul-loaded glycerosomal formulation had a cumulative drug penetration of 1591.50 ± 74.23 μg/cm^2^ across the nasal mucosa. In comparison, the Sul-loaded control in situ gel displayed a cumulative drug permeation of 466.85 ± 21.90 μg/cm^2^. Moreover, the glycerosomal in situ gel exhibited the highest J_ss_ value (238.27 μg/cm^2^·h) compared to the control in situ gel, which showed J_ss_ of 64.37 μg/cm^2^·h. This may potentially boost the permeability of Sul across the nasal membrane by utilizing an optimized glycerosomal gel (Er value of 3.72), hence promoting its permeation. The improved permeation of the optimized Sul-GMs in situ gel can potentially be related to the preparation’s constituents that disrupt the lipid surface barrier of the nasal mucosa. In addition, it is worth noting that the viscosity of the optimal preparation may have an impact on the absorption of the drug via the nasal mucosa [39]. The presence of glycerin can enhance the moisturizing and penetrating properties, hence facilitating the transportation of sulpiride across the nasal mucosa [40]. The effect of glycerin has the potential to enhance the efficacy of the glycerosomal gel formulation as a drug reservoir, facilitating the effective transportation of Sul through the nasal mucosa [41].

### 2.7. In Vivo Pharmacokinetic Study of the Optimized Sul-GMs

**Figure 9:**
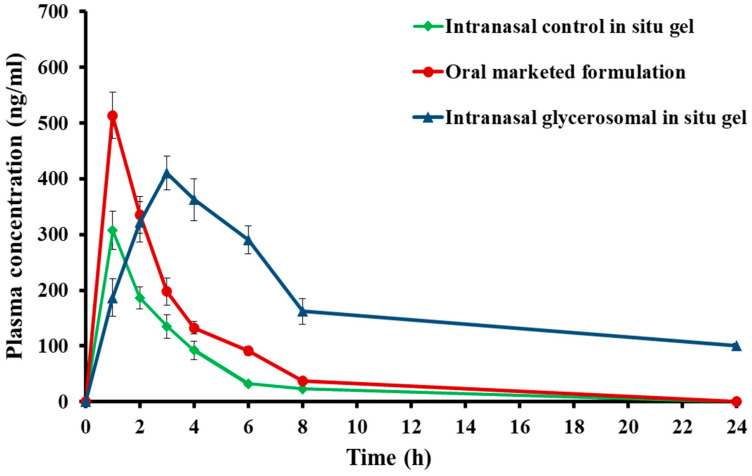
Plasma concentration–time curve of different Sul formulations.

The blood concentration–time profile of sulpiride after intranasal administration of the optimal formulation (G3) is shown in Figure 9 in comparison to that of intranasal administration of the control gel and oral commercially available product. The results showed that the optimized formulation had much higher plasma Sul concentrations than the control Sul in situ gel. 

Table 3 shows that the maximum concentration of Sul (C_max_) following intranasal delivery of the optimal in situ gel preparation (Sul-GMs) was 500.26 ± 32.01 ng/mL, which is significantly higher than the C_max_ obtained following application of the control gel (248.87 ± 27.46 ng/mL). Additionally, Table 2 demonstrates that the t_max_ of the Sul-loaded glycerosomal in situ gel formulation (Sul-GMs) was 6 h, the t_max_ of the control gel was 3 h, and the t_max_ of oral commercial product was 2 h. The optimum intranasal glycerosomal in situ gel showed a statistically significant increase in t_max_ compared to the other formulations studied, which may be indicative of the prolonged release mechanism of the glycerosomal in situ gel system. The use of prolonged release formulations has been shown to reduce unwanted effects and increase both therapeutic efficacy and bioavailability, as reported by Said and Elmenoufy [42]. In addition, the Sul-loaded glycerosomal in situ gel formulation had an AUC_0–t_ of 4540.27 ± 295.48 ng/mL^−1^·h. This result was statistically (*p* < 0.05) higher than both the in situ control gel (1179.45 ± 169.02 ng/mL^−1^·h) and the oral commercially available formulation (2388.41 ± 68.04 ng/mL^−1^·h). This may suggest that the intranasal glycerosomal gel system allows a slower Sul release than the oral marketed product and control gel, leading to a higher relative bioavailability of Sul. In addition, the half-life of the optimized glycerosomal in situ gel formulation was 12.04 ± 3.7 h, compared to 9.63 ± 1.34 h for the control gel and 5.41 ± 0.46 h for the oral marketed product. These results demonstrate the prolonged drug existence in the systemic circulation after administration of the optimum glycerosomal in situ gel formulation of Sul compared to the oral commercially available product and the control in situ gel. The optimized formulation had an MRT of 12.99 ± 3.97 h, while the control gel had an MRT of 11.28 ± 1.86 h, and the oral market product had an MRT of 4.79 ± 0.11 h. Intriguingly, Sul’s systemic bioavailability was significantly improved once it was included into a glycerosomal in situ gel. The relative bioavailability of the optimum formulation was around four times higher than that of the control gel and two times higher than that of the oral commercially available product. Based on the results, it shows that including Sul into glycerosomal in situ gels may greatly boost its systemic bioavailability compared to the other preparations studied.

## 3. Conclusions

Glycerosomes (GMs) are a special kind of liposomes that have been developed to contain high amounts of glycerin. Glycerosomes possess several advantages over liposomes, including enhanced stability and increased fluidity, which can be attributed to the high concentration of glycerin. The enhanced fluidity of GMs allows greater penetration into brain tissue compared to liposomes. The GMs loaded with sulpiride were created and then optimized using a BBD design to select the formulation with high encapsulation efficiency and maximum drug release. The optimum composition consisted of 497 mg of lipid and 31.13% *v*/*v* of glycerin. This formulation resulted in vesicles with 77.86 ± 1.24% entrapment efficiency and 61.83 ± 1.56% drug release after 6 h. Additionally, the transmission electron microscopy (TEM) analysis showed that the glycerosomal vesicles were spherical without any aggregates. Moreover, the optimal formula exhibited improved release compared to free drug suspension. The optimized formula was included into in situ gel bases. The glycerosomal in situ gel (Sul-GMs) preparations had satisfactory physical characteristics regarding viscosity, pH, and spreadability. Additionally, they significantly enhanced the ability of Sul to pass through the nasal mucosa. Notably, the in vivo pharmacokinetic experiments have shown that the optimal glycerosomal in situ gel preparation effectively improves the amount of Sul that reaches the bloodstream. This improvement is demonstrated by a fourfold rise in AUC_0–t_ when compared to the control gel and a twofold increase in comparison to oral product. In conclusion, Sul-GMs in situ gel could be a significant approach to improve the administration of Sul intranasally and to enhance its effectiveness for treating antipsychotic disorders.

## 4. Materials and Methods

### 4.1. Materials

Sulpiride (Sul) was acquired from Delta pharm. Co., Heliopolis, Egypt. Soy lecithin, cholesterol, absolute ethanol, glycerin, hydroxypropyl methyl cellulose-K15M, Carbopol 974P, and sodium azide were procured from Sigma Chemical Co. (St. Louis, MO, USA). 

### 4.2. Box-Behnken Design of Sulpiride-Loaded Glycerosomes

The Box–Behnken design was employed to investigate the impact of various formulation variables on the properties of sulpiride-loaded glycerosomes using Design Expert^®^ software (Ver. 7, Stat-Ease, Minneapolis, MN, USA). The independent variables were lipid amount (A), which varied from 250 to 500 mg, glycerin concentration (B), which lay between 10 and 40 *w*/*v* %, and time of sonication (C), which ranged from 10 to 30 min (Table 4). Consequently, there were a total of 15 trials conducted. The dependent variables were entrapment efficiency EE% (Y_1_) and in vitro drug release after 6 h (Y_2_). 

The analysis of variance (ANOVA) was conducted to determine the significance of each factor’s impact on the responds. The fitness of the data was evaluated by measuring various statistical items, such as the multiple correlation coefficient (R^2^) and the adjusted and predicted values. In addition, the adequacy of the explored models was assessed by examining the plots of predicted against actual results, graphs of residual versus experimental runs, and normal plots of residuals. A desirability function was employed to choose the optimal formula based on the maximum EE% (encapsulation efficiency) and in vitro drug release %.

### 4.3. Manufacturing of Sulpiride Glycerosomes

Glycerosomes loaded with sulpiride were generated using the thin film hydration method [28], employing lecithin as a lipid in a different concentration based on the obtained design. In a round-bottomed flask, the specified amounts of lecithin, cholesterol (20 mg), and sulpiride were dissolved in 10 mL of ethyl alcohol. A rotary evaporator (BÜCHI Labortechnik in Flawil, Switzerland) was employed to evaporate the organic solvent at a lowered pressure of 40 °C and 100 rpm until a lipid layer formed on the rounded bottom of the flask. Subsequently, a 10 mL solution of phosphate buffer with a pH of 7.4, containing varying amounts of glycerin, was employed, and the film was homogenized three times for five minutes.

### 4.4. Evaluation of Sulpiride-Loaded Glycerosomal Formulations

#### 4.4.1. Entrapment Efficiency %

The glycerosomal formulations were subjected to centrifugation at 10,000 rpm for one hour at a temperature of 4 °C using a centrifuge (TGL-16 Tabletop High Speed Refrigerated Centrifuge, Shanghai, China) in order to separate the glycerosomal vesicles from the sulpiride that was not encapsulated [24]. The nanovesicles obtained at the bottom of the centrifuge tube were rinsed with phosphate buffer at a pH of 6.4 and then subjected to centrifugation again to eliminate any drug that was not trapped inside. The process of rinsing nanovesicles was performed three times to guarantee the thorough elimination of any medication that was not encapsulated. The refined nanovesicles were retained for subsequent characterization. The sulpiride concentration in the supernatant was measured by diluting it appropriately and using a UV spectrophotometer at the specified λ_max_ of 293 nm [5]. The EE% was determined using the following equation:

$$EE\%=\frac{Total\;Sulpiride-Free\;Sulpiride}{Total\;Sulpiride}\;\times 100$$

#### 4.4.2. In Vitro Release Study of Sulpiride from the Generated Glycerosomal Preparations

This study investigated the release of sulpiride from the glycerosomal formulations in comparison to drug suspension. This was achieved by inserting the specified amount of the generated formulations into the dialysis bags with a molecular weight cut-off of 12,000–14,000 Da [43]. Subsequently, they were immersed in a 250 milliliter of phosphate buffer pH (6.4) at a temperature of 37 °C, while being agitated at 100 rpm. The quantity of sulpiride was estimated at various time intervals by collecting 5 mL from the dissolving media at 1, 2, 3, 4, 5, and 6 h and promptly replacing it with an equivalent volume of fresh media. Subsequently, the level of sulpiride in the obtained samples was measured using a UV spectrophotometer set at a wavelength of 293 nm. The tests were conducted three times, and the average percentage of sulpiride released at various time intervals was estimated.

### 4.5. Evaluation of the Optimum Sulpiride Formula

#### 4.5.1. Vesicles’ Size (VS) and Zeta Potential (ZP)

A Zetasizer Nano ZS instrument (Malvern Instruments, Worcestershire, UK) was utilized to determine the vesicle size of the optimized Sul-GMs after suitable dilution using distilled water at 25 °C [28]. The measurements were conducted in triplicate. 

#### 4.5.2. Transmission Electron Microscopy (TEM)

The morphology of the optimum formula was visualized using a transmission electron microscope (TEM; JEOL JEM-1010, Tokyo, Japan). Once the samples were appropriately diluted, they were placed onto a copper grid coated with carbon. Subsequently, the samples were coated with a 2% *w*/*v* solution of phosphotungstic acid. Then, the samples suspended in the air for a duration of 5 min in order to facilitate the drying process [44].

#### 4.5.3. Differential Scanning Calorimetry (DSC)

Differential scanning calorimetry (DSC) was conducted to analyze sulpiride, cholesterol, lecithin, and the optimal formulation. The equipment was used to heat samples containing approximately 5 mg at a rate of 10 °C/minute until an underflow of inert nitrogen at 200 °C was achieved [45].

### 4.6. Manufacturing of pH-Induced Carbopol Glycerosomal In Situ Gel

The pH-induced carbopol Sul-GMs in situ gels were manufactured utilizing HPMC K15M and Carbopol 974P. They were developed by adding HPMC to the glycerosomes after dispersion in distilled water. After that, the gelling agent (Carbopol) was incorporated, and the mixture was then left to hydrate overnight at 4 °C [46].

### 4.7. Characterization of pH-Induced Carbopol Glycerosomal In Situ Gel

#### 4.7.1. pH Value and Organoleptic Studies 

The pH of each formulation was detecting at room temperature using a pH meter (PCT-407 Portable pH Meter, Taipei City, Taiwan) [47]. Visual appearance and clarity were observed under fluorescent light against a white and black back ground for presence of any particulate matter.

#### 4.7.2. Viscosity Determination

Gel viscosity was measured by using Brookfield-R viscometer (Brookfield viscometer, Model DV-II, Middleboro, MA, USA). The apparatus was adjusted at 10 rpm at 37 ± 1 °C. 

#### 4.7.3. In Vitro Gelation Study and Gelation Capacity Determination

The determination of the gelation time of formulations was conducted following the procedures outlined by Sherafudeen and Vasantha [48]. The in situ delivery systems initially exist in a sol state prior to administration, but upon administration, they undergo a gelation process to transform into a gel. The evaluation of the carbopol formulations’ in vitro gelation capacity was carried out by adding a drop of the polymeric solution to 2 mL of PBS (pH, 6.4), a solution that simulates nasal fluid and is equilibrated at 37 °C. Next, the gel’s formation was visually assessed, and the required time for gelation and gel dissolving were recorded.

#### 4.7.4. Gel Strength Assessment

The gel strength was assessed by applying a certain weight (3.5 g) onto each gel sample (5 g) placed in a graduated measure. The gel’s strength was determined by measuring the time it took for a 3.5 g weight to move 5 cm downwards through the gel. [49]. The optimal time range for the nasal in situ gel was recorded as 25 to 50 s [35].

#### 4.7.5. Mucoadhesiveness Determination

The mucoadhesive strength of the generated formulations was assessed by conducting experiments on sheep nasal mucosa. A piece of sheep nasal mucosa was taken out of the animal’s nasal cavity and washed in saline and PBS with a pH of 6.4. The assessment of mucoadhesive strength was conducted utilizing a modified physical balance, as described by Salunke and Patil [50]. The right pan of the balance was substituted with an empty plastic cup. In place of the left pan of the balance, two glass vials linked base to base were utilized. The tested gel formulations (half gram) were sandwiched between two mucosal segments that had been soaked in pH 6.4 phosphate buffer saline at the base of each vial. The plastic cup was gradually filled with water until the two vials became detached from each other. The mucoadhesiveness, expressed as the detachment stress in dynes/cm^2^, was calculated using according to the following equation:

$$Detachment\;stress=\frac{Gravity\;acceleration\times weight\;of\;water\;in\;the\;plastic\;cup}{Area}$$

#### 4.7.6. Assessment of Spreadability

The spreadability of the gel was determined by distributing one gram of each gel sample in a circular shape with one centimeter diameter, using two glass plates placed opposite each other. Subsequently, a specific mass of 0.5 kg was exerted onto the plates. The sizes of the circles were altered due to the spreading of the gels, and these alterations were recorded [51]. 

### 4.8. Determination of the In Vitro Drug Release of Glycerosomal In Situ Gel

The in vitro release of glycerosomal in situ gels was evaluated utilizing the same procedures shown in Section 2.4.2.

### 4.9. Ex Vivo Drug Permeation Studies

The ex vivo drug penetration capabilities were investigated using fresh nasal tissue collected from the sheep nasal cavity. The mucosal membrane that had been cleansed was affixed to a locally fabricated diffusion cell that had a permeation area measuring 6.15 cm^2^. The glycerosomal in situ gel was placed within the donor segment. Samples were extracted from the acceptor segment, which contained 100 mL PBS (pH, 6.4) and 0.02% sodium azide, at specified time intervals and appropriately diluted for measuring drug absorbance at a wavelength of 293 nm. The steady-state drug flux (J_ss_) in units of μg/h·cm^2^ was determined by calculating the slope of the linear portion of the graph, which plotted the cumulative amount of drug penetrated per unit area (μg/cm^2^) against time [50]. Additionally, the enhancement ratio (Er) between the examined formula and the control formula (J_ss_ formula/J_ss_ control) was computed. The values were presented as the mean ± standard deviation (n = 3).

### 4.10. Bioavailability Study of the Optimized Glycerosomal In Situ Gel

#### 4.10.1. Study Design

Male Wistar albino rats weighing between 220 and 250 g were kept in controlled conditions of lighting, temperature, and relative humidity. Standardized pellet feed and clean drinking water were supplied to them. The Research Ethics Committee (REC) at the University of Ha’il, Saudi Arabia, approved all experiments conducted under Approval number, H-2023-361, on 19 September 2023. 

The study utilized a total of twenty-four rats. The animals were randomly divided into four groups, with each group consisting of six animals. The first group comprised rats that were used as control subjects and were provided with standard food. The second group consisted of animals that were administered the control in situ gel intranasally. The third group included animals that were administered the commercially available oral marketed formulation (Dogmatil^®^). The fourth group consisted of animals that received the optimized intranasal formulation containing Sul-loaded glycerosomal in situ gel. The oral marketed formulation (Dogmatil^®^), intranasal control in situ gel, and optimized intranasal glycerosomal in situ gel were all adjusted to achieve 15 mg Sul per kilogram of rat body weight [7]. Blood samples were obtained from the lateral tail vein at various time intervals (0, 1, 2, 3, 4, 6, 8, and 24 h) after treatment. The samples were collected in heparinized tubes to separate the plasma. The blood samples were subjected to centrifugation at 5000× *g* for a duration of 15 min. The plasma was isolated and preserved at a temperature of −20 °C for future utilization.

#### 4.10.2. HPLC Assay of Sulpiride 

The measurement of sulpiride in each sample was conducted at ambient temperature following the methodology established by Zidan et al. subsequent to modification and validation [2]. Samples of 500 μL of plasma were enriched with 25 μL of internal standard (paracetamol 100 μg/mL) and 0.1 mL of a sodium hydroxide solution (1 N) using vortex mixing for two minutes. The samples were mixed with 4 mL of ethyl acetate/dichloromethane (3:1, *v*/*v*), then the mixed solutions were vortex for one minute followed by centrifuge for the sample for 10 min at 6000 rpm. Finally, the supernatants were transferred into centrifuge tubes and evaporated under a nitrogen stream then reconstituted in 1 mL mobile phase into an auto-sampler vial and injected into the HPLC system. The mobile phase was prepared by adding one gram of hexane sulphonic acid to 900 mL water, the solution was shaken, and then the volume was completed to 1000 mL with water and pH adjusted to 3. The prepared solution was mixed with acetonitrile, 80:20, *v*/*v*. The HPLC system comprised a 2690 Alliance HPLC system equipped with a Waters 996 photodiode array detector. The HPLC analysis was conducted at room temperature with a flow rate of 1 mL/min and injection volume of 100 μL. The effluent that was produced by the column was observed using spectrophotometry at a wavelength of 254 nm.

#### 4.10.3. Pharmacokinetic Parameters

A graph depicting the average sulpiride plasma concentrations throughout time was created. The non-compartmental technique, aided by the PKsolver tool in Microsoft Excel, was utilized to compute the plasma pharmacokinetic parameters. Pharmacokinetic characteristics involve the maximum concentration of a drug in the plasma (C_max_, ng/mL) as well as the time it takes to achieve this peak concentration (T_max_, hour). In addition, the trapezoidal rule was used to calculate the area under the curve (AUC) from time zero to 24 (AUC_0–24_, ng mL^−1^ h) and from time zero to infinity (AUC_0–∞_). Furthermore, the elimination half-life (t_1/2_) and the mean residence time (MRT) were computed. The calculation of relative bioavailability was also performed.

### 4.11. Analysis of Statistical Data

ANOVA was used for statistical analysis of data. This study was conducted via GraphPad Prism^®^ version 5.00.

## Figures and Tables

**Table 1:** Different glycerosomal preparations and the corresponding results of dependent factors.

Formulation	Independent Variables			Dependent Variables	
	A (mg)	B (%)	C (min)	Y_1_ (%)	Y_2_ (%)
F1	375	40	10	57.43 ± 1.09	49.41 ± 2.78
F2	500	25	10	79.32 ± 1.00	59.97 ± 2.38
F3	500	25	30	63.98 ± 0.99	60.94 ± 0.86
F4	250	25	10	47.98 ± 0.50	38.74 ± 0.73
F5	250	40	20	38.87 ± 0.71	35.38 ± 1.63
F6	250	25	30	55.60 ± 1.02	43.36 ± 2.21
F7	375	25	20	65.96 ± 1.91	54.02 ± 1.35
F8	375	40	30	49.18 ± 1.89	47.94 ±1.46
F9	250	10	20	41.81 ± 0.67	29.74 ± 2.22
F10	500	40	20	70.07 ± 0.69	50.91 ± 1.97
F11	375	25	20	64.92 ± 2.09	53.74 ± 0.92
F12	375	25	20	65.63 ± 1.20	54.44 ± 0.76
F13	375	10	10	46.78 ± 2.52	44.61 ± 1.04
F14	375	10	30	47.51 ± 1.87	51.33 ± 0.53
F15	500	10	20	49.98 ± 0.65	51.80 ± 0.75

A—amount of phospholipid (mg); B—glycerin concentration (%); C—sonication time (min); Y_1_, EE (%); Y_2_, In vitro release (%).

**Table 2:** Different sulpiride-loaded glycerosomal in situ gel formulations.

Formulation	Gel Base	HPMC Concentration (% *w*/*w*)	Gelation Capacity	pH	Spreadability (cm)	Gel Strength (s)	Mucoadhesive Strength (dyne/cm^2^)	Viscosity (cP)
G1	Carbopol 974P (0.2% *w*/*w*)	0.2	-	ND	ND	ND	ND	ND
		0.4	+	6.36 ± 0.17	4.62 ± 0.08	14.37 ± 0.78	1949.43 ± 14.64	5848.30 ± 43.93
		0.6	+	6.48 ± 0.09	4.38 ± 0.11	19.67 ± 0.86	2365.57 ± 20.05	6096.70 ± 60.15
		0.8	++	5.79 ± 0.19	4.22 ± 0.58	23.57 ± 0.95	2572.77 ± 29.81	6784.97 ± 38.48
		1.0	+++	6.30 ± 0.15	4.02 ± 0.13	27.80 ± 0.26	2723.87 ± 27.54	7204.93 ± 64.79
G2	Carbopol 974P (0.4% *w*/*w*)	0.2	-	ND	ND	ND	ND	ND
		0.4	+	6.42 ± 0.19	4.42 ± 0.08	12.67 ± 0.76	2721.20 ± 14.70	7163.6 ± 44.10
		0.6	++	6.49 ± 0.17	4.25 ± 0.10	19.37 ± 0.91	2889.47 ± 24.60	7725.17 ± 73.81
		0.8	+++	6.07 ± 0.14	4.08 ± 0.12	35.27 ± 0.70	3030.23 ± 18.74	8090.70 ± 56.22
		1.0	+++	5.89 ± 0.08	3.82 ± 0.10	38.50 ± 0.83	3282.73 ± 10.90	8848.20 ± 32.71
G3	Carbopol 974P (0.6% *w*/*w*)	0.2	+	5.79 ±0.19	3.93 ± 0.15	11.53 ± 0.45	3188.24 ± 16.49	8711.73 ± 36.39
		0.4	++	6.30 ± 0.15	3.68 ± 0.08	21.77 ± 0.49	3549.23 ± 14.93	9090.70 ± 56.22
		0.6	++	6.38 ± 0.13	3.47 ± 0.06	31.03 ± 0.55	3927.50 ± 33.21	9848.20 ± 32.70
		0.8	+++	6.40 ± 0.09	3.22 ± 0.10	42.03 ± 0.45	4175.90 ± 27.93	10,046.40 ± 34.48
		1.0	+++	6.07 ±0.12	3.02 ± 0.13	52.13 ± 0.32	4682.133 ± 11.49	10,148.07 ± 36.30

HPMC, hydroxypropyl methyl cellulose; (-): no gelation; (+): gelation occurs within a short period of time and afterwards disperses quickly; (++): the process of gelation occurs rapidly and persists for a duration of 12 h; (+++): the gelation process occurs promptly and persists for a duration exceeding 12 h; (ND): not determined, no gel formation.

**Table 3:** Pharmacokinetic parameters of different Sul formulations.

Parameters	Oral Marketed Product	Control Sul Gel	Optimized Sul-GMs Gel
C_max_ (ng/mL)	698.56 ± 42.06	248.87 ± 27.46	500.26 ± 32.01
t_max_ (h)	2	3	6
K_el_ (h^−1^)	0.13 ± 0.01	0.07± 0.01	0.062 ± 0.02
t_1/2_ (h)	5.41 ± 046	9.63 ± 1.34	12.04 ± 3.7
AUC_0–t_ (ng/mL^−1^·h)	2388.41 ± 68.04	1179.45 ± 169.02	4540.27 ± 295.48
MRT (h)	4.79 ± 0.11	11.28 ± 1.86	12.99 ± 3.97
Relative bioavailability (%)	202.50	-	384.85%

**Table 4:** BB design for sulpiride-loaded glycerosomes optimization.

Independent Variables	Type	Actual Levels	
		Low	High
A: Lipid amount (mg)	Numeric	250	500
B: Glycerin concentration *v*/*v* (%)	Numeric	10	40
C: Time of sonication (Minutes)	Numeric	10	30
**Response Variables (Dependent Variables)**	**Goal**		
(Y_1_) = Encapsulation efficiency EE%	Maximize		
(Y_2_) = In vitro release of the drug after 6 h	Maximize		

## Data Availability

The original contributions presented in the study are included in the article, further inquiries can be directed to the corresponding author.
